# Mn-Doped BaTiO_3_ Ceramics: Thermal and Electrical Properties for Multicaloric Applications

**DOI:** 10.3390/ma12213592

**Published:** 2019-10-31

**Authors:** Alexander Semenov, Antonina Dedyk, Ivan Mylnikov, Oleg Pakhomov, Andrey Es’kov, Alexander Anokhin, Vasiliy Krylov, Anton Burovikhin, Yulia Pavlova, Alexander Tselev, Andrei Kholkin

**Affiliations:** 1 Department of physics, Saint Petersburg State Electrotechnical University, St. Petersburg 197376, Russia; semalexander@gmail.com (A.S.); dedyk_ai@mail.ru (A.D.); mylnikov.il@gmail.com (I.M.); antonburovihin@mail.ru (A.B.); yulia.pavlova@gmail.com (Y.P.); 2 Laboratory “Materials and Structures for Electro- and Magnetocaloric Energy Conversion”, ITMO University, St. Petersburg 197101, Russia; oleg.cryogenics@gmail.com (O.P.); aeskow@gmail.com (A.E.); itmo.tfi@gmail.com (V.K.); atselev@ua.pt (A.T.); 3 SCAMT Institute, ITMO University, St. Petersburg 197101, Russia; sanman4242@gmail.com; 4 Department of Mathematics and Physics, Lappeenranta University of Technology, 53850 Lappeenranta, Finland; 5 Department of Physics & CICECO-Aveiro Institute of Materials, University of Aveiro, 3810-193 Aveiro, Portugal; 6 School of Natural Sciences and Mathematics, Ural Federal University, Ekaterinburg 620000, Russia

**Keywords:** BaTiO_3_, electrocalorics, magnetocalorics, phase transition, specific heat

## Abstract

Multiferroic materials are widely used in microelectronics because they are sensitive to elastic, magnetic, and electric fields and there is an intrinsic coupling between them. In particular, transition metal-doped BaTiO_3_ is considered as a viable multiferroic because of the simultaneous presence of ferroelectricity and magnetism. In this work, we study the electrical and thermal properties of Mn-doped BaTiO_3_ ceramics that can be used for multicaloric applications. We found that Mn doping leads to the broadening and shifting of the phase transition accompanied with simultaneous decrease of latent heat and entropy. Mn doping causes a decrease in the bulk resistivity while contact resistance remains intact. Doped ceramics can withstand high electric fields (up to 40 kV/cm) and exhibit linear I-V characteristics followed by the Schottky limited current in contrast to earlier observations. As such, these ceramics are promising for multicaloric applications.

## 1. Introduction

In the last decade, there has been a revival of interest in the study of multiferroics [1,2], which have a great potential for the development of magnetoelectric [3], magnetooptic [4] and multicaloric [5] devices for solid-state cooling. Indeed, strong coupling between ferroelectric and magnetic order parameters in these materials, for which external magnetic field induces a change of the ferroelectric characteristics, and electric field generates a change in magnetization, would allow development of a wide range of technological innovations, including memory media with ferroelectric writing and magnetic reading, sensors of magnetic field, energy harvesting devices and solid-state refrigerators. However, known single-phase magnetic ferroelectrics usually have low magnetic ordering temperatures [6,7], thus limiting the possibilities for their use. One way to increase magnetoelectric coupling is doping of ferroelectric antiferromagnetic perovskites such as BiFeO_3_ with both A- and B-site impurities that either induce magnetism via exchange interaction or strongly distort the antiferromagnetic state, so as to release the spatially modulated spiral magnetic moment [8]. The highest values of the spontaneous magnetization have been observed for the rare-earth-doped bismuth ferrite [9,10], in which strong contribution from magnetic moments of the rare-earth ions to the net magnetization takes place. Moreover, for diamagnetic A-site doped BiFeO_3_, spontaneous magnetization has been shown to significantly increase [11,12]. 

Another strategy is to dope non-magnetic ferroelectrics such as BaTiO_3_ with transition metal ions like Fe or Mn that could develop the magnetic moment in the perovskite structure. Moreover, large recoverable electrostrain after the aging treatment in Mn-doped BaTiO_3_ ceramics has been observed that is promising for stress-induced magnetism [13,14]. Although doping of BaTiO_3_ has been intensively investigated so far. Most work has focused on either its electric or magnetic properties and did not consider thermal properties that are important for multicaloric cooling [15,16,17,18,19]. In one report [20] particles of undoped BaTiO_3_ were coated with Mn-doped barium titanate by a co-precipitation method and then sintered to form Mn-modified BaTiO_3_ ceramics. Consequent measurement of unipolar piezoelectric strain demonstrated that the increase in the hysteresis was suppressed with Mn-doping. Madhan et al. [21] synthesized Mn-doped BaTiO_3_ ceramics by sol–gel combustion method. Both dielectric permittivity and AC conductivity were improved with Mn doping. Mn-doped BaTiO_3_ ceramics were also prepared by the conventional solid-state reaction route [22]. Dielectric loss decreased with the increasing Mn content, however, doping had no significant effect on the average grain size of BaTiO_3_.

In this work, 0, 5 and 10 at. % Mn-doped BaTiO_3_ ceramics sintered via conventional mixed oxide route have been investigated in view of using them in caloric applications. Specific heat, electric conductivity at a high electric field (up to 40 kV/cm), and dielectric permittivity have been studied in a wide temperature range (250–500 K). These results provide clear prospects for using these ceramics for multicaloric applications, however, the properties must be improved to withstand a higher electric field.

## 2. Materials and Methods

Undoped and Mn-doped BaTiO_3_ (BTO) ceramics were produced by the conventional mixed oxide route [16] followed by sintering in air. The starting oxides were mixed according to their stoichiometric ratios and sintered at 1350 °C. Barium carbonate (BaCO_3_), titanium dioxide (TiO_2_) and manganese dioxide (MnO_2_) were used as starting materials. The raw materials were purchased from the NevaReaktiv company (St. Petersburg, Russia). Two-stage synthesis was performed, with intermittent grinding and granulation of the samples. Pure BTO ceramic samples had a high relative density (up to 94%) close to values of theoretical densities. The densities of Mn-doped samples were substantially lower (up to 83%). Doping was performed with 5 (BTO+5Mn) and 10 at. % Mn (BTO+10Mn). The grain size of the ceramics was in the range 0.1–1 μm. The dielectric parameters and DC electric conductivity were measured in plane-parallel ceramic disks with silver paste electrodes. The plate thickness was varied within the range h = 0.25–1.35 mm with a disk area S = 25–120 mm^2^.

Temperature measurements of the sample capacitance were performed with an Agilent E4980A precision LCR meter (Keysight Technologies, USA). A sample in the form of a plate with electrodes was fixed in a special holder placed in a climatic chamber. The measurements were performed in the temperature range from 200 to about 500 K with the temperature variation rates of 0.01 to 20 K/min. The capacitance measurement precision was 0.05%. The specific heat was measured in aluminum crucibles with DSC 204 Phoenix F1 (NETZSCH, Germany) differential scanning calorimeter at heating and cooling rates of 20 K/min in the temperature range 240–455 K. The heat capacity measurement error was less than ±3%.

To examine the electrical conductivity at room temperature, current-voltage (I-V) characteristics of plane-parallel capacitor structures were measured under electric fields up to 40 kV/cm. The current was measured with a V7-30 electrometer in the range 10^−13^–10^−5^ A with the accuracy of less than 10%.

Local domain structure was mapped by the piezoresponse force microscopy mode installed in the commercial atomic force microscope MFP-3D (Asylum Research, Oxford Instruments, UK).

## 3. Results and Discussion

### 3.1. Crystallograhic and Domain Structure

An analysis of the doped samples by the transmission electron microscopy (TEM) demonstrated that the pseudocubic perovskite phase (pure BaTiO_3_) is the main phase, with two manganese-containing phases also present.

The main Mn-containing phase is Ba(Mn_x_Ti_1–x_)O_3_ in which Mn ions substitute Ti^4+^ ions and cause the appearance of O^2−^ vacancies. However, there are also grains with monoclinic secondary phase Ba_4_(Mn_x_Ti_1–x_)_12_O_27_. An X-ray fluorescence microanalysis was used to determine the real content of Mn in these phases (Table 1). The grain size of the ceramics was in the range 0.1–1 μm in BTO+10Mn samples, with a smaller average size, observed for BTO+5Mn. As such, doping was inhomogeneous, and some grains did not contain Mn at all, whereas others were expected to be magnetic, i.e., multiferroic composite structure was realized. Composite multiferroics are the most promising for magnetoelectric coupling, in which it is mediated by the intergranular mechanical stress exerted by ferroelectric grains on the magnetic ones [3]. The valence state of Mn in magnetic grains could be beneficial for exchange coupling and the existence of a non-zero magnetic moment [19].

**Figure 1:**
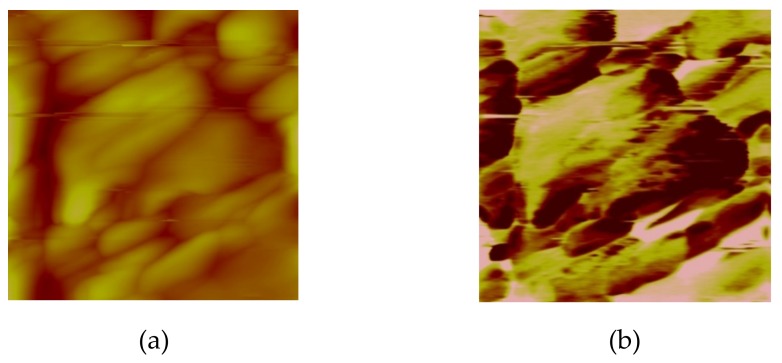
(**a**) Topography and (**b**) piezoresponse force microscopy image (amplitude) of BTO+5Mn ceramics demonstrating inhomogeneous polarization distribution. The image size is 5 × 5 µm^2^.

The results clearly demonstrate that some grains have a distinct piezoelectric response, while some of them do not exhibit any polarization at all. This can be related to the centrosymmetric character of Ba_4_(Mn_x_Ti_1–x_)_12_O_27_ phase, which is not supposed to be piezoelectric. Similar contrast was observed in BiFeO_3_ ceramics doped with rare earth elements in which coexistence of polar and non-polar grains could be revealed by PFM in the broad composition range [23]. This is expected for the composite structures, however, more detailed analysis (e.g., a combination of magnetic force microscopy and electron backscatter diffraction microscopy is required to confirm this scenario [24]). At this time, we can just conclude that bigger grains seem to exhibit stronger contrast as compared to smaller grains in BTO+5Mn ceramics (Figure 1). 

### 3.2. Dielectric Permittivity vs. Temperature Dependences

**Figure 2:**
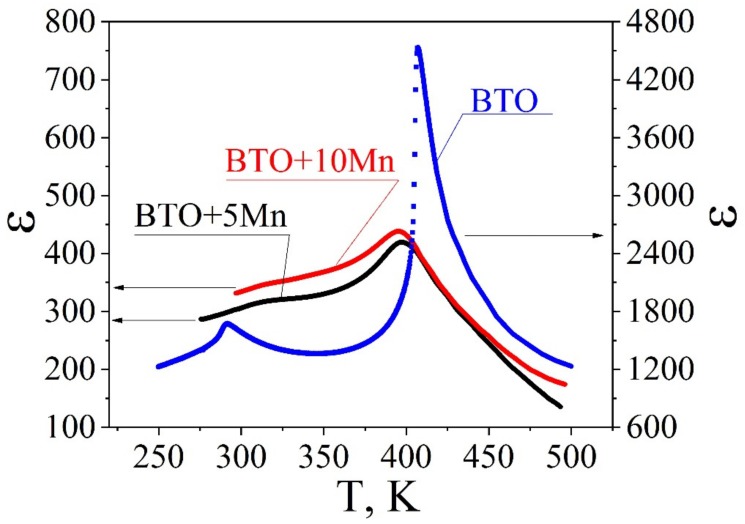
Temperature dependences of the effective dielectric permittivity of pure BTO ceramics (**blue curve**) in comparison with that of Mn-doped samples. Much lower values of the permittivity in BTO-5Mn (**black curve**) and BTO-10Mn (**red curve**) are due to non-ferroelectric grains of the second phases.

Figure 2 shows a comparison of the temperature dependences of the relative dielectric permittivity ε, calculated based on the capacitance vs. temperature curves in pure BTO ceramics and those with two Mn concentrations. The characteristic abrupt change in the relative permittivity of pure barium titanate at Tm = Tc = 407 K unambiguously indicates the onset of the first-order phase transition from cubic to tetragonal phase. At a lower temperature (~287.6 K), the phase transition to the rhombohedral phase was observed, with its position corresponding to previously published data [25,26]. In what follows, we consider and discuss only the temperature maximum corresponding to the cubic-to-tetragonal phase transition, at T ~400 K. With increasing Mn concentration, the maximum temperature T_m_ decreases by 10–12 K. The ε values for Mn-doped samples much lower (about 450). This is a natural result of the composite structure of the samples and low permittivity value of the monoclinic Ba_4_(Mn_x_Ti_1–x_)_12_O_27_ phase [27]. However, the ferroelectric character of the BaTiO_3_ grains is conserved even in highly doped samples as evidenced by their piezoelectric contrast and dielectric anomalies at the phase transition.

### 3.3. Specific Heat as a Function of Temperature and Doping

**Figure 3:**
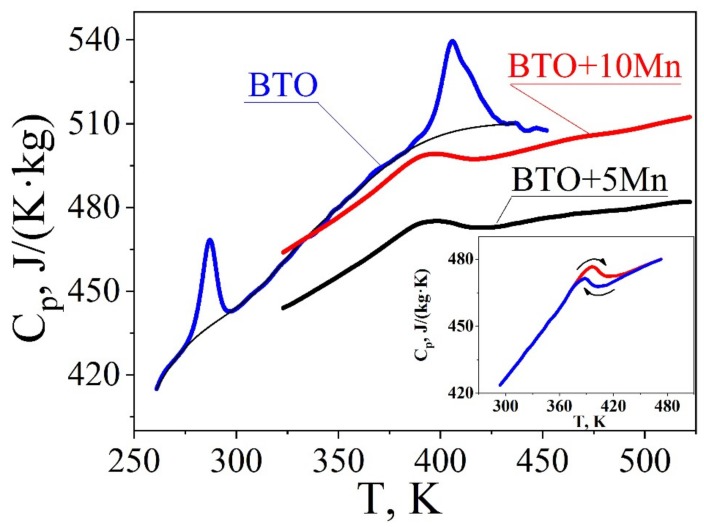
Specific heat capacity in pure BTO ceramics (**blue curve**) in comparison that of Mn-doped samples. The inset shows the Cp in heating and cooling cycles for lightly doped BTO (5% Mn).

Figure 3 shows the temperature dependences of the heat capacity (specific heat at constant pressure) of the studied samples, Cp(T). The temperatures at which the specific heat is at maximum corresponds to dielectric anomalies (Tm) within 1–2 K. The value of the heat capacity and its temperature dependence for pure BTO are close to the published data [26,27,28]. In Mn-doped samples, specific heat is substantially lower and the anomaly at the cubic-to-tetragonal phase transition is smoother. However specific heat is almost recovered in the 10% doped samples. The inset to Figure 3 compares temperature dependences of the heat capacity for the sample doped with 5 mol % Mn measured under cooling and heating. It can be seen the phase transition temperature is 5 K higher on heating, compared with that undercooling, which is characteristic of the first-order phase transition [29]. Similar dependence was also observed near the cubic-to-tetragonal phase transition for 10 mol % Mn samples. We calculated the excess heat (ΔQ) and entropy (ΔS) at the phase transition according to the formula

(1) $$\Delta S=\int_{T_{2}}^{T_{1}}\frac{\Delta C}{T}dT,$$

where T_1_ and T_2_ are the integration temperatures and ΔC is a jump of specific heat at the phase transition.

The entropy change is one of the most important parameters for the electrocaloric energy conversion because it governs the temperature change in the adiabatic cycle. Heat capacity has to be also known because the temperature change in the electrocaloric effect can be evaluated by the indirect method [30]

(2) $$\Delta T_{EC}^{ID}=-T\int \frac{1}{C_{E,\sigma }}{\left(\frac{\partial P}{\partial T}\right)}_{E,\sigma }dE.$$

It is seen that the effective Curie–Weiss constant describing thermodynamical properties of the ceramics drops by about an order of magnitude in Mn-doped samples, while the excess heat is only two times smaller, as expected for composite samples with a significant content of non-ferroelectric phase. However, the specific heat for composite samples also decreases with Mn addition, so that the reduction of excess heat (according to Equation (2)) can be partly compensated.

### 3.4. Conductivity at a High Electric Field

**Figure 4:**
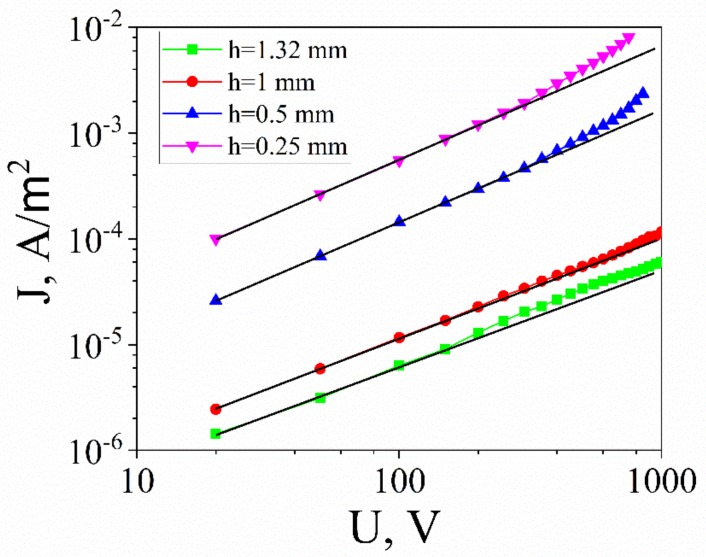
Current density of the platelet capacitors of BTO+10Mn ceramics. The evaluation of the bulk and contact resistance was done based on linear fit (**black solid lines**).

**Figure 5:**
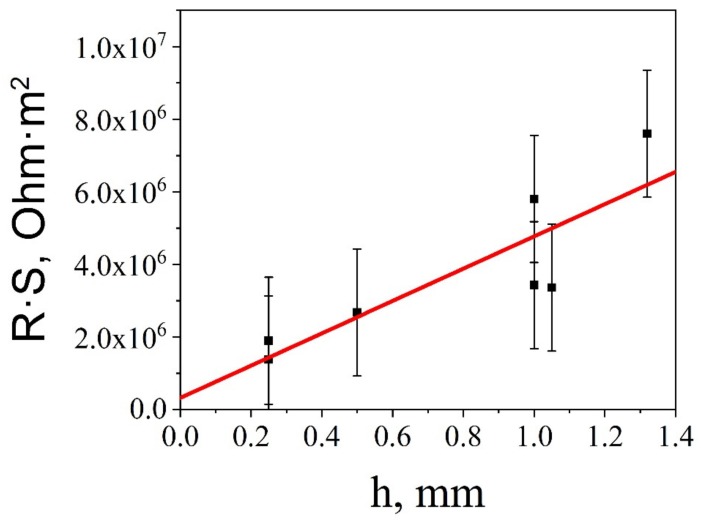
Total resistance times surface area of BTO+10Mn ceramics as a function of the thickness of ceramic discs. The intersection with the ordinate axis yields the contact resistance of the sample.

Since electrocaloric phenomena occur under a high electric field applied to the sample, the I-V characteristics have to be studied and contact and bulk resistivities have to be evaluated. At these field strengths, the I-V characteristics of most of the insulators become nonlinear. We performed the measurements of the current under different voltages for various thicknesses for all samples, as exemplified in Figure 4 for the BTO-10Mn sample. The Ohmic part of the I-V characteristic (up to E ≈ 5 × 10^4^ V/m) was used to estimate the bulk resistance, which decreased with increasing Mn concentration from ~10^12^ Ohm for pure BTO to ~10^10^–10^11^ Ohm for doped samples (for the thickness h ≥ 1 mm). The bulk resistivity was evaluated by the formula:

(3) $$R\cdot S=\frac{h}{\sigma }+R_{k}$$

where R is the total resistance, R_k_ is the contact resistance (in series with the bulk), h is the thickness, and σ is the bulk conductivity of the ceramics. Figure 5 shows the experimental dependence R·S(h) vs. h. Upon its approximation with LSM, the result of extrapolation of the resulting straight line makes it possible to determine the unit area resistance Rc of the contact region and bulk electrical conductivity of the ceramics. For the (BTO+10Mn) ceramics, the electrical conductivity was σ = (2 ± 2) × 10^−10^ Ω^−1^·m^−1^. The values of resistances of the ceramics are compared in Table 2. Non-linear part of the I-V characteristics in metal-dielectric-metal structures is commonly associated with the following mechanisms: Schottky barrier emission from the metal electrodes, field emission from the deep traps, and Poole–Frenkel effect. To choose the type of the conduction mechanism we plotted measured I-V characteristics in different coordinates [31,32], i.e., ln(J) = f(E^1/2^) for the Schottky emission, ln(J/E^2^) = f(1/E) for the emission from traps, and ln(J/E) = f(E^1/2^) for the Poole–Frenkel effect. Rigorous analysis of the non-linear portions of the I-V characteristics carried out in terms of the above-mentioned mechanisms demonstrates that the highest values of the Pearson fitting criterion (χ = 0.99903–0.99969) corresponds to the conductivity mechanism associated with the Schottky emission.

### 3.5. Discussion

Doping with Mn ions leads to smoothening of the phase transition and a shift of the transition point to lower temperatures, as compared with pure BTO ceramics (Table 2). Such a shift is an evidence of the decreased stability of the ferroelectric phase and contradicts to the literature data in which an increase of the transition temperature was observed [16]. It could be due to the increase of disorder as well as a consequence of the mechanical stress exerted by the non-ferroelectric phase. However, even in highly doped ceramics, the anomalies in the dielectric permittivity vs. temperature dependences are clearly visible. The results of combined measurements of the heat capacity and dielectric permittivity suggest that a first-order phase transition is observed. Taking into account that specific heat hysteresis in doped samples occurs under heating and cooling near the phase transition and small values of the excess entropy (Table 2), we can assume that this phase transition is more like a displacement-type transition. The small values of the excess entropy of the phase transition are commonly attributed to displacement type phase transition [29]. The excess heat (ΔQ) and entropy (ΔS) of phase transitions were determined for these ceramics for the first time. For samples with 10 mol % Mn, these values somewhat exceed those for the samples with 5 mol % Mn, but are substantially smaller than those for the pure BTO ceramics. In accordance with the thermodynamic theory of ferroelectricity, the gain in entropy at the phase transition is due to the change in the spontaneous polarization [16,23]:

(4) $$\Delta S=\frac{P^{2}}{2C\rho \varepsilon_{0}}.$$

Using this expression we can estimate the spontaneous polarization of Mn-doped ceramics, and these values are presented in Table 2. The calculated value of spontaneous polarization for pure BTO agrees well with that reported in the literature [26,27,28]. However, the results obtained via direct measurements of the spontaneous polarization (not shown here) give a value that is an order of magnitude smaller than that obtained from thermal measurements. The same discrepancy between the polarization estimated by pyroelectric effect and dielectric measurements has been observed for other ferroelectric materials [28,29]. Estimates of the polarization for the doped samples, (BTO+5Mn) and (BTO+10Mn), provide the values that are three to five times smaller than those for pure BTO (Table 2). However, this polarization is quite sufficient for the application of heavily doped ceramics for multicaloric cooling applications [33,34].

## 4. Conclusions

In this work, we studied electrical and thermal properties of heavily Mn-doped BaTiO_3_ ceramics that is intended for further use in multicaloric applications. We found that the Mn doping leads to the broadening and shifting of the phase transition accompanied by the simultaneous decrease of latent heat and entropy. In addition, Mn doping causes a strong decrease in the bulk resistivity while contact resistance remains intact. Doped ceramics can withstand high electric fields (up to 40 kV/cm) and exhibit linear I-V characteristics followed by the Schottky limited type conduction in contrast to earlier observations. As such, these ceramics have the potential to be used in multicaloric applications.

## Figures and Tables

**Table 1:** Phases in the investigated samples.

Sample	Main Phases	Mn Content
BTO	Tetragonal phase BaTiO_3_	-
BTO+5Mn	Tetragonal phase BaTiO_3_ Monoclinic phase Ba_4_Ti_12_O_27_	Ba(Mn_0.02_Ti_0.98_)O_3_ Ba_4_(Mn_0.12_Ti_0.88_)_12_O_27_
BTO+10Mn	Tetragonal phase BaTiO_3_ Monoclinic phase Ba_4_Ti_12_O_27_	Ba(Mn_0.03_Ti_0.97_)O_3_ Ba_4_(Mn_0.14_Ti_0.86_)_12_O_27_

**Table 2:** Curie–Weiss constant K, transition temperature Tc, density ρ, excess heat ΔQ, entropy change ΔS, remanent polarization P, and resistance R in pure BTO, Mn-doped BTO, and BTO single crystals.

Composition of Ceramic Samples	K, K	T_c_, K	ρ, kg/m^3^	ΔQ, J/kg	ΔS, J/kg·K	P, C/cm^2^	R, Ω
BTO	0.88 × 10^5^	407.7	5.65 × 10^3^	584	1.434	11.2 × 10^−6^	10^12^
BTO+5M	0.8 × 10^4^	397	5.05 × 10^3^	246	0.620	2.1 × 10^−6^	4 × 10^10^
BTO+10M	0.15 × 10^5^	395	4.65 × 10^3^	258	0.653	2.83 × 10^−6^	2.4 × 10^11^
BaTiO_3_ single crystal [28]		401		899	2.24	(16 − 24) × 10^−6^	
BaTiO_3_ single crystal [24]	1.7 × 10^5^		6.02 × 10^3^		2.15	20 × 10^−6^	
